# Application of bio-layer interferometry for the analysis of ribosome-protein interactions

**DOI:** 10.3389/fmolb.2024.1398964

**Published:** 2024-08-01

**Authors:** Ilamathy Pandiarajan, Sujata B. Walunj, Nirjhar Banerjee, Janmejaya Rout, Sanjeeva Srivastava, Swati Patankar, Sandip Kaledhonkar

**Affiliations:** Department of Biosciences and Bioengineering, Indian Institute of Technology Bombay, Mumbai, India

**Keywords:** ribosome-protein interaction, bio-layer interferometry, binding affinity, translation factors, ribosome

## Abstract

The ribosome, a ribonucleoprotein complex, performs the function of protein translation. While ribosomal RNA catalyzes polypeptide formation, several proteins assist the ribosome throughout the translation process. Studying the biochemical and kinetic properties of these proteins interacting with the ribosome is vital for elucidating their roles. Various techniques, such as zonal centrifugation, pull-down assays, dynamic light scattering (DLS), fluorescence polarization, and surface plasmon resonance (SPR) are employed for this purpose, each presenting unique advantages and limitations. We add to the repertoire of techniques by using Bio-Layer Interferometry (BLI) to examine interactions between the ribosome and translation factors. Our findings demonstrate that BLI can detect interactions of *Escherichia coli* ribosomes with two proteins: *E. coli* initiation factor 2 (IF2) and *P. falciparum* translation enhancing factor (PTEF). A protein (Green Fluorescent Protein; GFP) known not to bind to *E. coli* ribosomes, shows no binding in the BLI assay. We show that BLI could be used to study the ribosome-protein interactions as it has key advantages like label-free procedures, ease of assay performance, and ribosome sample reuse. Our results highlight the comprehensive use of BLI in studying the ribosome-protein interactions, in addition to studying protein-protein and protein-ligand interactions.

## 1 Introduction

The ribosome serves as the protein synthesis machinery of the cell. A prokaryotic ribosome comprises two subunits – the small subunit (30S) and the large subunit (50S) jointly weighing ∼2.5 MDa (Nomura, 1970). This macromolecular complex consists two-thirds of ribosomal RNA, which catalyzes polypeptide formation, and one-third ribosomal proteins, that acts as a scaffold (Wilson and Nierhaus, 2005). Protein translation occurs in three stages: i) initiation, ii) elongation, and iii) termination and recycling (Schmeing and Ramakrishnan, 2009). Along the course of translation, several proteins also known as translation factors, aid the ribosome in a stage-specific manner to accomplish protein synthesis (Grigoriadou et al., 2007; Goyal et al., 2015; Wasserman et al., 2016; Adio et al., 2018). Additionally, various proteins are involved in translation regulation by associating with the ribosome during its biogenesis (Connolly et al., 2008), cellular stress (Ueta et al., 2008; Polikanov et al., 2012), protein rescue and quality control (Safdari et al., 2022). Binding studies of these proteins with the ribosome are essential to decipher their intricate roles in these important cellular processes. Investigations into ribosome-protein interactions also aid in understanding the involvement of ribosomal proteins in causing ribosomopathies (Aspesi and Ellis, 2019) and contribute to drug development (Kang et al., 2021).

Classically, ribosome-protein interactions were studied using zonal centrifugation (Hershey et al., 1969; Fakunding and Hershey, 1973), co-sedimentation (Moreno et al., 1998), and pull-down assays (Spencer and Spremulli, 2005). In all these assays, the detection of ribosome interaction with protein(s) has been studied using either radioactive scintillation (Hershey et al., 1969; Fakunding and Hershey, 1973; Caserta et al., 2006) or immunoblotting (Moreno et al., 1998; Spencer and Spremulli, 2005). These techniques consume large amounts of ribosome samples in volume and concentration. Translation factors that induce subunit association (Godefroy-Colburn et al., 1975; Antoun et al., 2004) or dissociation (Coatham et al., 2015) are studied using dynamic light scattering (DLS). The DLS technique is employed when a considerable size change is monitored, such as during ribosome subunit association or dissociation. However, it is not suitable when significant size differences exist between two biomolecules, as the binding of one molecule may not bring about any detectable change in the size of the monitored species. Fluorescence polarization provides a solution to this limitation as it is not constrained by particle size. In fluorescence polarization, the translation factors are fluorescently derivatized and then monitored for binding to ribosomes to obtain quantitative binding affinity (Weiel and Hershey, 1982). To study the spatial and dynamic binding properties of the proteins interacting with the ribosome, Förster Resonance Energy Transfer (FRET) is employed. This method is useful in elucidating detailed translation mechanisms (Milon et al., 2010; Goyal et al., 2015) and requires fluorescent labeling of both the binding partners. More recently, label-free methods such as Surface Plasmon Resonance (SPR) have been beneficial in studying proteins interacting with the ribosome (Li et al., 2009; Benedix et al., 2010; Wu et al., 2012). These binding studies are important in identifying novel translation factors involved in protein translation. They offer preliminary insights before advancing to structural investigations, which are essential for understanding the spatial organization and the detailed mechanisms of these translation factors in protein translation.

Here, we evaluate a known technique – “Bio-Layer Interferometry (BLI)” – that has not been reported to study ribosome-protein interactions to the best of our knowledge. BLI is a preferred method to study the interaction between biomolecules due to the requirement of relatively low volume and concentration (nanomolar range) of samples (Sultana and Lee, 2015). Other advantages are that BLI is a less labor-intensive technique that provides instantaneous binding details of biomolecules under study and it is a medium to high-throughput technique depending on the instrument used (Tobias and Kumaraswamy, 2021). Generally, BLI is used to investigate protein-protein (Sultana and Lee, 2015; Horbowicz-Drożdżal et al., 2021), protein-ligand (Han et al., 2018; Afsar et al., 2022), and protein-DNA (Barrows and Van Dyke, 2022) interactions. In this report, we utilize BLI for studying the ribosome-protein interaction, leveraging the mentioned advantages.

**FIGURE 1:**
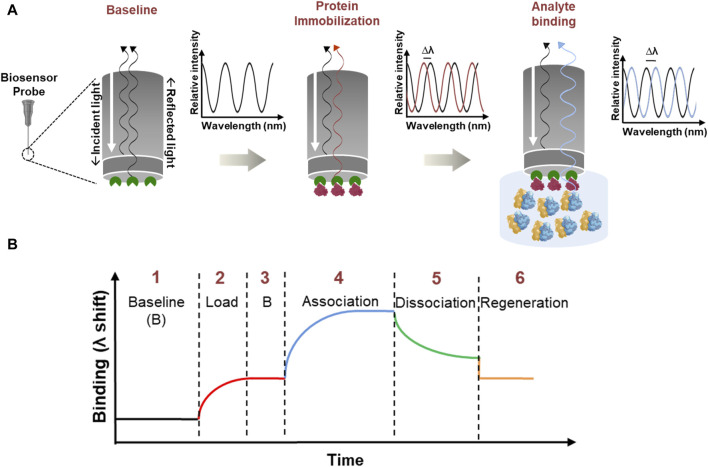
Illustration of Bio-Layer Interferometry. **(A)** Stepwise schematic which shows the BLI principle with an enlarged view of the biosensor probe which has a streptavidin coating shown in green color. *Immobilization*: the biotinylated protein (red) binds to the streptavidin coating (green) on the biosensor probe with a strong biotin-streptavidin interaction. *Analyte binding*: the immobilized probe is dipped into an analyte solution (blue) containing ribosomes. If there is a specific interaction between the ribosomes and the immobilized protein, the ribosomes will bind to the protein, forming a complex. *Optical Signal Shift*: as the ribosome binds to the immobilized protein, the thickness of the biolayer at the probe interface increases. This change in thickness affects how the white light passes through the tip and generates an interference pattern. The instrument detects this shift in the interference pattern as a signal, indicating a successful binding between the analyte and the immobilized protein. **(B)** General sensorgram of a binding event. BLI involves a series of processes: (1) baseline stabilization, (2) protein immobilization (load), (3) stabilization, (4) association, (5) dissociation, and (6) regeneration. To perform kinetic analysis, steps 3–6 are repeated.

BLI is a label-free, optical biosensing technique that applies the “Dip and Read” methodology to calculate the interaction between biomolecules (Sultana and Lee, 2015). An illustration of BLI and the steps involved is provided in Figure 1. In principle, white light passed along the biosensor probe is analyzed for a shift in the interference pattern between an optical reference layer and the biolayer (Nirschl et al., 2011). Here, the ligand (one of the biomolecules under study) is immobilized to the biosensor probe and is dipped into the analyte solution (containing the other biomolecule of interest). An interaction between the biomolecules leads to an increase in optical thickness, eventually causing a shift in the interference pattern (Nirschl et al., 2011). A series of kinetic experiments identify the real-time binding interactions and provide quantitative parameters like on, and off rates (*k*_*on*_, *k*_*off*_ respectively) and binding affinity (K_d_) (Tobias and Kumaraswamy, 2021). These quantitative binding parameters help to elucidate the nature and intensity of binding events between the biomolecules.

We used the *Escherichia coli* initiation factor (IF2), a well-known translation factor involved in 70S ribosome initiation complex (70S IC) formation, to validate the BLI technique (Sprink et al., 2016). We successfully demonstrated the binding of purified *E. coli* initiation factor (IF2) with its 70S ribosome using BLI techniques, thus showing, for the first time as far as we know, its utility in understanding the ribosome-protein interaction. Another protein that has been shown to interact with *E. coli* 70S ribosomes is the *P. falciparum* translation enhancing factor (PTEF) (Chan et al., 2017). Our BLI protocol shows that PTEF also binds to purified *E. coli* 70S ribosomes. In contrast, a protein that is known to show no interactions with *E. coli* 70S ribosomes (Green Fluorescent Protein; GFP), does not bind in the BLI assays. This report adds to the list of techniques that can be used to study protein interactions with the ribosome, laying the path for BLI to be included in the repertoire of assays to study this biologically important macromolecular complex.

## 2 Materials and methods

### 2.1 Strains and plasmids

*E. coli* Rosetta strain was used for ribosome isolation. The plasmid containing *E. coli* IF2 was a kind gift from Dr. Debasis Das, TIFR Mumbai. The codon-optimized *P. falciparum* PTEF-CTD (756–1,192 amino-acids) was gene synthesized and cloned into a pET43a + vector (GenScript). The GFP containing pET28a plasmid was available in our lab (Babar et al., 2016). Growth of the strains was carried out in LB media (HiMedia).

### 2.2 Extraction and purification of *E. coli* 70S ribosome

Purified 70S ribosomes were obtained from *E. coli* Rosetta cells as specified in previously published protocols (Blanchard et al., 2004). Concisely, *E. coli* Rosetta cells were cultured until OD_600_ nm reached 0.8. The cell pellets were resuspended in the buffer system containing 10 mM Mg^2+^ concentration as described by Blanchard et al., 2004. The cell pellets were sonicated and the lysate obtained was clarified at 10,500 g for 1 h, 4°C (SIGMA 3-30KS centrifuge). The clarified lysate was layered onto Tris buffer containing 1.1 M sucrose and centrifuged at 112,000 *g* (Rotor: Type 70 Ti, Beckman Coulter: Optima XPN 100), 22 h, 4°C to obtain crude ribosome. To obtain 70S ribosome, the crude ribosome preparation was layered onto a 20%–50% sucrose gradient and centrifuged at 70,000 *g* (Rotor: SW-32 Ti, Beckman Coulter) for 17 h, 4°C. The fractions containing 70S ribosomes were monitored using A_260_ nm readings (JASCO V-730) and then concentrated using a 100 kDa protein concentrator (Thermo-Scientific).

### 2.3 Expression and purification of proteins

*E. coli* IF2 was purified as previously explained by (Shimizu et al., 2001) with modifications. *E. coli* IF2 was expressed in *E. coli* BL21 DE3 strain under the following induction conditions: 0.5 mM IPTG, 18°C, 16 h. Cells were resuspended in binding buffer (20 mM HEPES pH 7.5, 100 mM KCl, 10 mM MgCl_2_, 3 mM βME, Pierce™ Protease Inhibitor Mini Tablet (Thermo-Scientific)). The clarified supernatant was incubated with Ni-NTA agarose beads (Genetix) for 2 h and elution was done using increasing imidazole concentration. Fractions that had IF2 bands were collected, dialyzed with buffer (20 mM HEPES pH 7.5, 100 mM KCl, 10 mM MgCl_2_, 3 mM βME), and concentrated using Pierce™ Protein Concentrator PES (30 kDa MW cut-off).

GFP was expressed using the following induction conditions: 0.5 mM IPTG, 16°C, 16 h (Babar et al., 2016). Sodium phosphate buffer solution was used for resuspension of pellets and incubated with Ni-NTA agarose beads (Genetix) for 2 h. Increasing imidazole concentrations were used to elute the GFP protein. GFP-containing fractions were collected, dialyzed with storage buffer (20 mM HEPES pH 7.5, 100 mM KCl, 10 mM MgCl_2_, 3 mM βME), and concentrated using a Pierce™ Protein Concentrator PES (10 kDa MW cut-off).

The codon-optimized *P. falciparum* PTEF-CTD (756–1,192 amino-acids) was gene synthesized and cloned into a pET43a + vector (GenScript). The purification of PTEF-CTD was done as previously mentioned (Chan et al., 2017). Briefly, the PTEF-CTD containing pET43a + plasmid was expressed in *E. coli* BL21 DE3 strain with induction conditions as follows: 0.5 mM IPTG, 3 h, 37°C. Resuspension of cell pellets was done in lysis buffer (10 mM HEPES pH 7.5, 150 mM NaCl, 5% glycerol, 10 mM imidazole, 3 mM βME, Pierce™ Protease Inhibitor Mini Tablet (Thermo-Scientific)). The clarified lysate was applied to the Ni-NTA Agarose beads (Genetix) for 3 h and elution was done stepwise increasing the concentration of imidazole. The fractions containing PTEF-CTD were dialyzed with storage buffer (20 mM HEPES pH 7.5, 150 mM NaCl, 10 mM MgCl_2_, 3 mM βME), and concentrated using Pierce™ Protein Concentrator PES (30 kDa MW cut-off).

### 2.4 Bio-layer interferometry assay

#### 2.4.1 Biotinylation of bait/ligand

“Bait” or “ligand” is the biomolecule that is immobilized onto the biosensor probe. These biosensor probes have various surface chemistry such as streptavidin, amine reactive groups, or Ni-NTA coating (Tobias and Kumaraswamy, 2021). In our study, High Precision Streptavidin (SAX) biosensor tips (Part No. 18-5117, Sartorius) were used for the immobilization of ligand molecules. The protein samples (*E. coli* IF2, GFP, *P. falciparum* PTEF-CTD) and *E. coli* 70S ribosomes were all biotinylated using EZ-link NHS-LC-LC-Biotin (Thermo-Scientific) and incubated in ice for 2 h. The ligand-to-biotin molar ratio for biotinylation was used as follows: 1:10 for proteins and 1:20 for the ribosome. Following biotinylation, the mixture was desalted using Zeba Spin columns (0.5 mL volume) with a 7K molecular weight cut-off. Around 100 µL of the biotinylation mixture was added to the spin column and centrifuged at × 2,000 g for 2 min. The eluate obtained contained the biotinylated ligand while the spin column retained the unbound biotin. The column was subsequently rinsed with five column volumes of 1x PBS and stored at 4°C for future use.

#### 2.4.2 Instrumentation

**FIGURE 2:**
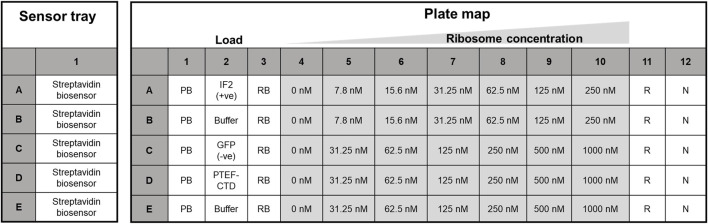
Sensor plate and assay plate layout used for kinetic analysis. The Streptavidin biosensor tips in the sensor tray (on the left) were hydrated in protein buffer, while the right panel housed the plate map for kinetic analysis. Biotinylated proteins (*E. coli* IF2, GFP, and PTEF-CTD) were loaded in lane two for ligand immobilization, and row B and E of the 96-well plate served as the reference sample lane. Here, PB, protein buffer, RB, ribosome buffer, R, regeneration buffer, and N, neutralization buffer, respectively.

The Octet RED96e system from ForteBio (Sartorius) was used. It is a multi-channel system that can handle eight samples in parallel (Tobias and Kumaraswamy, 2021). Two 96-well plates (opaque, flat-bottom, Greiner Bio-One) were used – one served as the sensor plate and the other as the sample plate with the layout as shown below in Figure 2. The sensor tray, plate outline, and step timing were defined using Octet BLI® Discovery Software. The biosensor tip moved across the 96 well plate with core steps: i) Biosensor tip hydration ii) Ligand immobilization/Loading iii) Baseline stabilization iv) Association v) Dissociation and vi) Regeneration of biosensor tip. Their time course is tabulated in Table 1, with the data collection rate set to 2 Hz. The plates were shaken at 1,000 rpm and maintained at 30 °C along the course of the experiment.

**TABLE 1:** Tabulation of time course for each step in BLI assay. Octet BLI® Discovery software was used to define the time course of each step.

Step name	Step time (seconds)
Baseline	60
Baseline 0	600
Association	1,200
Dissociation	600
Regeneration	5
Loading	1,000

#### 2.4.3 Reagent composition

Ribosome buffer (Kinetic buffer) consisted of 20 mM Tris HCl pH 7.5, 60 mM NH_4_Cl, 7.5 mM Mg(CH_3_COO)_2_, 0.5 mM EDTA, and 6 mM βME. BSA (0.1%) and Tween-20 (0.02%) were freshly added to the ribosome buffer to remove non-specific binding and to prevent the blocking of unoccupied sites on the biosensor tip by analyte components which can adversely affect the binding affinity calculation between two biomolecules (Sultana and Lee, 2015; Tobias and Kumaraswamy, 2021).

Protein buffer (IF2 and GFP) consisted of 20 mM HEPES pH 7.5, 100 mM KCl, 10 mM MgCl_2_, and 3 mM βME.

Protein buffer (PTEF-CTD) consisted of 20 mM HEPES pH 7.5, 150 mM NaCl, and 3 mM βME. Both protein buffers were utilized as the equilibration and the neutralization buffer.

*E. coli* IF2 was incubated with GTP analog, GDPNP (Sigma Aldrich), in a 1:1,000 M ratio and incubated at 37°C for 10 min before the start of the experiment.

The regeneration buffer was made of 10 mM glycine pH 2.5.

#### 2.4.4 Steps involved in the kinetic assay

Hydration of biosensor tip: The high-precision Streptavidin biosensor tips were hydrated by placing them on the sensor plate containing protein buffer before the start of the experiment for 10 min.

Ligand Immobilization: The biosensor tip after hydration was dipped into lane two for ligand immobilization. Lane two had 0.25 µM load (*E. coli* IF2, GFP, *P. falciparum* PTEF-CTD and buffer only respectively) row-wise. Generally, 10 μg/mL–50 μg/mL of protein is considered a good range for ligand immobilization (Sultana and Lee, 2015).

Baseline stabilization: Lanes one and three had protein buffer which was used for signal stabilization. The immobilized biosensor tip was dipped into lane three to check for signal stabilization. The initial baseline stabilization step had 600 s to check for stable ligand immobilization and the subsequent baseline stabilization step was for 60 s.

Kinetic assay: Lanes 4 – 10 had increasing concentrations of the *E. coli* 70S ribosome as shown in Figure 2. The kinetic buffer contained BSA (0.1%) and Tween 20 (0.02%) to reduce non-specific binding (Tobias and Kumaraswamy, 2021). The concentration of BSA (0.1–1 mg/mL), Tween 20 (0.01%–0.09%) is considered a good range for the kinetic experiments performed (Sultana and Lee, 2015; Tobias and Kumaraswamy, 2021). The ligand-immobilized biosensor tip was dipped into the analyte solution to check for association and back into lane three to monitor dissociation. We used 1,200 s for association and 600 s for dissociation. This was followed by a regeneration step.

Regeneration step: As the biosensor tip must be used for varying analyte concentrations, it was important to remove the bound analyte. Lane 11 had regeneration buffer (10 mM glycine, pH 2.5) which removes the bound analyte interacting with the ligand immobilized to the biosensor tip (Tobias and Kumaraswamy, 2021). This was done for 5 s, 3 times in total, for each concentration.

Neutralization step: The biosensor probe which was regenerated was then dipped into the protein buffer present in lane 12 for equilibration. This was done for 5 s, 3 times in total, for each concentration. The biosensor probe was then subjected to continue from the baseline stabilization step onwards for the next analyte concentration.

#### 2.4.5 Data analysis of kinetic assay

Octet® Analysis Studio 12.2.2.26 was used for preprocessing, where the data points obtained were double-referenced, negating the wavelength shift with the reference biosensor well and with the reference sample well. This was done to reduce the error due to baseline shift and to reduce the non-specific binding of analyte components to streptavidin tips (Tobias and Kumaraswamy, 2021). To obtain the binding affinity (K_d_), the equilibrium responses at varying analyte concentrations were fitted using the following equation $Y=\frac{B_{\max}*X}{K_{d}+X}$ . Here, Y is the equilibrium response for different analyte concentrations, B_max_ refers to maximum response and X represents the different analyte concentrations used.

### 2.5 Fluorescence anisotropy

The intrinsic fluorescence of GFP was utilized for anisotropy experiments. The excitation (λ_ex_ = 475 nm) and emission spectra (λ_em_ = 510 nm) for GFP (0.25 µM) was identified using JASCO FP-8350 spectrofluorometer. FP-8550 JASCO spectrofluorometer was used for performing anisotropy experiments with the above chosen emission and excitation wavelength. The anisotropy measurements were done at room temperature with 5 nm excitation and emission slit width. The response was measured at 0.5 s with three accumulations. Free GFP (0.25 µM) was loaded in a cuvette with a pathlength of 1 cm to obtain the anisotropy. Later varying *E. coli* 70S ribosome concentrations (31.25 nM, 62.5 nM, 125 nM, 250 nM, 500 nM and 1,000 nM) were titrated with GFP (0.25 µM) to obtain the anisotropy values. Free *E. coli* 70S ribosome was also checked for polarization at an emission wavelength of 510 nm.

### 2.6 Negative staining of ribosome samples and visualization in TEM

The purified ribosome samples were applied to carbon-coated copper grids (CF200-CU, 200 mesh, EMS). The sample was allowed to be adsorbed for 1 min and was washed with buffer to remove excess sample. To the grid, 2% uranyl acetate was added for 10 s, blotted to remove excess stain, and dried. The grids were visualized using a 300 kV transmission electron microscope (Themis 300 G3, Thermo-Scientific).

## 3 Results

### 3.1 Ribosomes do not show stable loading on the biosensor tip

Before starting the experiments, we confirmed that the ribosomal preparation was clean with negative staining using transmission electron microscopy (Supplementary Figure S1) and that the protein preparations showed purified proteins of the correct size, with minimal non-specific bands (Supplementary Figure S2).

**FIGURE 3:**
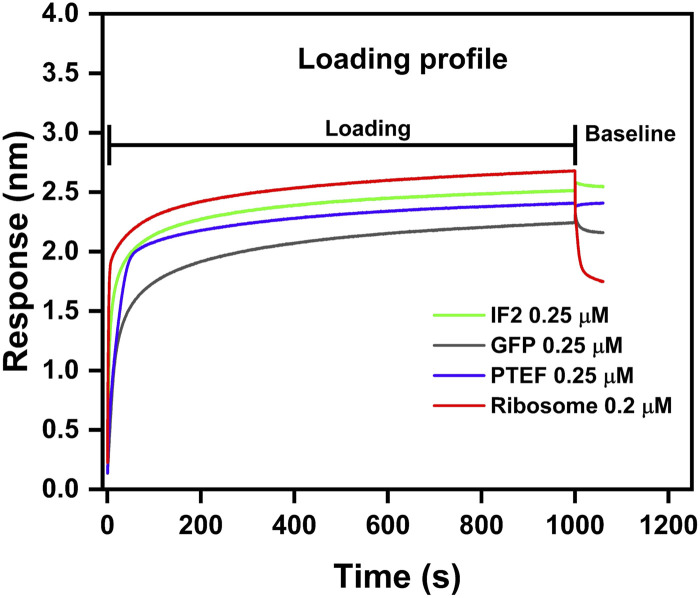
Loading profile comparison between IF2, GFP, PTEF-CTD, and *E. coli* 70S ribosome. *E. coli* IF2 (green), GFP (grey), and PTEF-CTD (blue) had 0.25 µM load respectively; *E. coli* 70S ribosome (red) had 0.2 µM load. The loading step was carried for 1,000 s which was followed by 600 s of baseline stabilization.

We assessed the ligand immobilization profile of both biotinylated proteins and ribosomes to the Streptavidin biosensor tips. Loading of biosensor tips with both biotinylated proteins (0.25 µM each of IF2, PTEF-CTD, and GFP) and biotinylated ribosome (0.2 µM) resulted in a significant wavelength shift, confirming immobilization (Figure 3). The loading profile of the ribosome preparation onto the sensor tips resulted in an observable wavelength shift, even with a minimal load (0.2 µM) (Figure 3). However, when the tips were subsequently dipped into the buffer well to check for stabilization of the loading response, the ribosome immobilized tip displayed a decrease in wavelength shift (Figure 3). This could be attributed to the ribosome dissociating from the biosensor tip due to its large size (∼2–4 MDa). On the other hand, the protein immobilization was successful and stabilized, allowing for further kinetic experiments to be performed.

### 3.2 Validation of BLI technique

**FIGURE 4:**
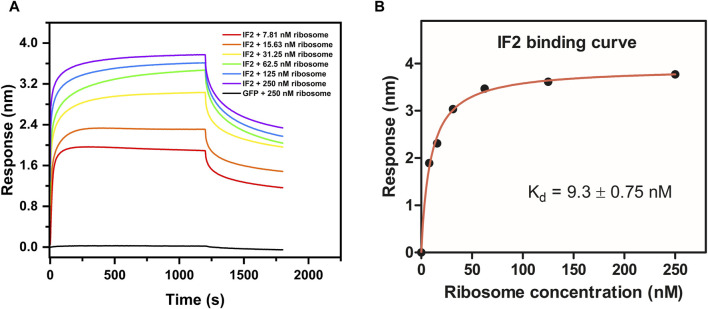
Determination of binding affinity (K_d_) between *E. coli* IF2 and *E. coli* 70S ribosome. **(A)** Binding sensorgram of *E. coli* IF2 (0.25 µM) with *E. coli* 70S ribosome (7.8 nM, 15.6 nM, 31.25 nM, 62.5 nM, 125 nM, and 250 nM). Negative control GFP (0.25 µM) with 250 nM of *E. coli* 70S ribosome is represented in black. **(B)** Binding affinity curve between *E. coli* IF2 and *E. coli* 70S ribosome.

The validation of ribosome-protein interaction was carried out by assaying the binding between 70S ribosomes and *E. coli* initiation factor IF2. *E. coli* IF2 was incubated with the GTP analog (GDPNP) at 37°C for 10 min to prevent the dissociation of the 70S ribosome once bound to it (Sprink et al., 2016). The 70S ribosome served as the analyte and the *E. coli* IF2*GDPNP immobilized tip was dipped into varying analyte concentrations. The shift in interference pattern was observed with increasing concentrations of the 70S ribosome (Figure 4A) which indicated that the *E. coli* 70S ribosome was bound to *E. coli* IF2. The binding affinity calculated for three replicate experiments (Figure 4B; Supplementary Figures S3B, S3D) is provided in Table 2 and the average binding affinity was found to be 4.5 ± 0.78 nM.

**TABLE 2:** Tabulation of binding affinity (K_**d**_) obtained using BLI studies. The binding affinity was calculated with the following equation: $Y=\frac{B_{\max}*X}{K_{d}+X}$ . Here, the maximum response during association (B_max_) was plotted against varying analyte concentrations to obtain binding affinity (K_d_).

Experiments	Replicates	Binding affinity K_d_ _(nM)_	*R* ^2^
*E. coli* IF2 with *E. coli* 70S ribosome	n = 1	9.3 ± 0.75	0.997
	n = 2	2.7 ± 0.17	0.999
	n = 3	1.5 ± 0.21	0.998
*P. falciparum* PTEF-CTD with *E. coli* 70S ribosome	n = 1	426 ± 80.4	0.989
	n = 2	312.6 ± 61.53	0.985
	n = 3	515.4 ± 3.59	0.999

We used GFP as a negative control. Studies using co-immunoprecipitation have shown that GFP does not interact with ribosomes (Chan et al., 2017). No interaction was visible when titrating 0.25 µM biotinylated GFP against varying concentrations of the *E. coli* 70S ribosome (Figure 4A). GFP binding to the *E. coli* 70S ribosome was also examined using fluorescence anisotropy experiment to confirm that there is no binding between GFP and 70S ribosomes (Supplementary Figure S4). Hence, the BLI technique could be employed to investigate specific proteins that bind to the ribosome with precision.

### 3.3 Interaction of PTEF-CTD with *E. coli* 70S ribosome

**FIGURE 5:**
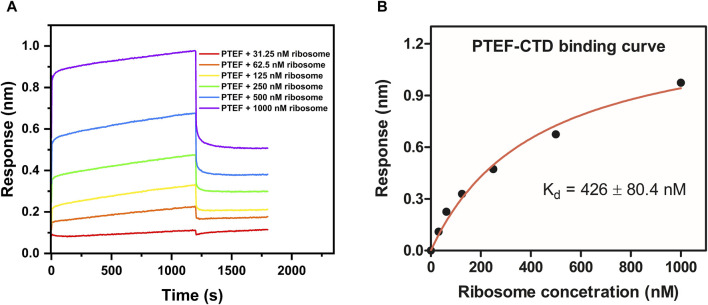
Determination of binding affinity (K_d_) between *P. falciparum* PTEF-CTD and *E. coli* 70S ribosome. **(A)** Binding sensorgram of PTEF-CTD to *E. coli* 70S ribosome (31.25 nM, 62.5 nM, 125 nM, 250 nM, 500 nM, and 1,000 nM). **(B)** Binding affinity curve between PTEF-CTD and *E. coli* 70S ribosome.

The C-terminal domain of PTEF has a SAM-like domain which is an RNA-binding domain (Chan et al., 2017). Previously, an *in vitro* reconstituted translation assay had shown functional, qualitative interactions of *E. coli* 70S ribosome with *P. falciparum* PTEF-CTD (Chan et al., 2017). Therefore, the BLI technique was used to check for the binding of *P. falciparum* PTEF-CTD to the *E. coli* 70S ribosome. The shift in interference pattern was monitored as the PTEF-CTD immobilized biosensor tip moved across the analyte solution (*E. coli* 70S ribosome) (Figure 5A). The analysis demonstrated binding between PTEF-CTD and 70S ribosome as shown in Figure 5A. The binding affinity for three replicate experiments (Figure 5B; Supplementary Figure S4D) is provided in Table 2 and the average binding affinity was found to be 0.42 ± 0.114 µM. Thus, using BLI we were able to show the binding of PTEF-CTD with the *E. coli* 70S ribosome.

## 4 Discussion

In this study, we introduce the use of BLI as an alternate technique to study ribosome-protein interactions, for the first time to our knowledge. Though several techniques like ultracentrifugation, DLS, and FRET are available to date to study ribosome-protein interactions (Benedix et al., 2010; Milon et al., 2010; Coatham et al., 2015), BLI served to be a simple, user-friendly, real-time, and medium-throughput technique (Sultana and Lee, 2015). Additionally, BLI allows for the recovery and reuse of samples used during the assay, providing an added advantage for samples such as ribosomes that must be purified from large volumes of bacterial cultures and thus have constraints in their large-scale purification (Tobias and Kumaraswamy, 2021). Table 3 provides a summary of the existing techniques used to study ribosome-protein interaction, including BLI.

**TABLE 3:** Summary of techniques used to study ribosome-protein interactions.

Methods	Measurement	Sample volume	Concentration	Rate constants kon, koff	Binding affinity kd	Cost	Sample throughput	Labelling	Sensitivity range
Zonal Centrifugation	Indirect	Comparatively high	Ribosome: µM range Ligand: 0.1–10X Kd	No	Yes	Minimal	User-dependent	No	mM to µM
Pull-down assay	Indirect	Comparatively high	Ribosome: µM range Ligand: 0.1–10X Kd	No	Yes	Minimal	User-dependent	No	mM to µM
Dynamic light scattering	Real-time	Depends on instrumentation	Ribosome: µM range Ligand: 0.1–10X Kd	Yes	Yes	Instrumentation cost	Instrument dependent	No	mM to pM
Fluorescence polarization	Real-time	Depends on instrumentation	Tracer: µM range Ligand: 0.1–10X Kd	No	Yes	Instrumentation cost	Instrument dependent	Fluorescent labelling	mM to pM
FRET	Real-time	Depends on instrumentation	Ribosome: µM range Ligand (0.1–10X K_d_)	Yes	Yes	Instrumentation cost	Instrument dependent	Fluorescent labelling	mM to fM
Surface Plasmon Resonance	Real-time	Less (∼400 µL)	Ligand: 2–50 μg/mL Analyte: 0.1–10X Kd	Yes	Yes	Instrumentation and biosensor chip	2 samples per biosensor chip (Biacore T200)	Biotin-Streptavidin, specific antibodiesetc.	1 mM to 10 fM (Biacore T200)
Bio-Layer Interferometry	Real-time	Less (∼200 µL)	Ligand: 10–50 μg/mL Analyte: 0.1–10X Kd	Yes	Yes	Instrumentation and biosensor tip	8 samples per biosensor tip (OCTET RED96e)	Biotin-Streptavidin, specific antibodiesetc.	1 mM to10 p.m. (Octet RED96e)

This report presented a detailed methodology for using the BLI technique in studying ribosome-protein interactions. We used a positive control (*E. coli* IF2 – 98 kDa), negative control (GFP – 28 kDa), and an unknown (*P. falciparum* PTEF-CTD – 55 kDa) to check for their binding against *E. coli* 70S ribosome. Our study indicated that smaller protein molecules mentioned above served as better load than larger ribosome molecules (∼2.5 MDa). This was evident from Figure 3, where the biotinylated ribosomes dissociated from the biosensor tip during the baseline stabilization process. Also, due to its substantial size, the ribosome causes increased thickness at the biolayer during a binding event, leading to significant wavelength shift (Figure 3). Monitoring large wavelength shifts is much easier, making it preferable to use small molecules as load and large molecules as analytes (Nirschl et al., 2011; Sultana and Lee, 2015).

In our kinetic experiments, the first baseline step was extended for 600 s, during which the protein-immobilized biosensor tip was dipped into the kinetic buffer. The time duration for the first baseline step was intentionally extended compared to subsequent baseline steps to ensure proper signal stabilization (Table 1). Also, the kinetic buffer contained non-specific binding inhibitors like BSA and Tween-20 which prevented the binding of analyte components to the streptavidin biosensor tips. It was essential to add these non-specific binding inhibitors for accurate binding affinity calculation.

Two proteins, namely, *E. coli* initiation factor (IF2) and *P. falciparum* PTEF, were studied for their interaction with *E. coli* 70S ribosomes using BLI. *E. coli* IF2, which is involved in 70S ribosome initiation complex formation (Sprink et al., 2016; Kaledhonkar et al., 2019), displayed binding to *E. coli* 70S ribosomes. We also determined the binding of PTEF-CTD with the *E. coli* 70S ribosome, which was previously shown to interact with the *E. coli* ribosome (Chan et al., 2017). In contrast, GFP was shown not to bind to *E. coli* 70S ribosomes, ascertaining the precision of BLI in identifying proteins with specific interactions with ribosomes.

While the BLI technique can detect proteins interacting with the ribosome, it also has limitations. Techniques like BLI and SPR, in principle, immobilize a ligand molecule, which can impede the orientational possibility for analyte binding (Sultana and Lee, 2015). The amount of biotinylated protein immobilized on an SAX biosensor tip is not a fixed value for a particular concentration and the surface density of streptavidin coating on the tip that could vary across the lot (Weeramange et al., 2020). Hence, even if three replicate experiments with similar reaction conditions were performed, each of these experiments must be considered separately for binding affinity calculation (Table 2). While BLI offers a convenient way to study ribosome-protein interactions using label-free procedures and no prior knowledge of binding sites, it has limitations. It can provide initial estimates of binding strength, but these need confirmation with other methods like fluorescence polarization or FRET. However, compared to the latter mentioned methods, BLI has advantages like user-friendliness, label-free technique, and the ability to recover and reuse the samples after the experiment.

Abundant knowledge exists regarding translation factors interacting with the ribosome. However, several other proteins interacting with the ribosome are continually being discovered in different cell types and human pathogens, whose roles have not yet been deciphered. Bio-Layer Interferometry (BLI) could be employed to study the interaction between these speculated proteins and the ribosome, thus shedding light on their involvement in biogenesis, stress management, and rescue mechanisms occurring in the organism. Furthermore, the role of ribosomal proteins in ribosome-associated diseases or ribosomopathies could be elucidated using BLI. As the ribosome is historically a successful drug target, these studies will eventually aid in drug development.

## Data Availability

The raw data supporting the conclusions of this article will be made available by the authors, without undue reservation.
