# Supplementary material for: Application of bio-layer interferometry for the analysis of ribosome-protein interactions
Source: Front Mol Biosci. 2024 Aug 1;11:1398964. doi: 10.3389/fmolb.2024.1398964 (PMC11325027; doi:10.3389/fmolb.2024.1398964)
Supplement: Supplementary file 1 [file DataSheet1.docx]

**Supplementary Material**

**Supplementary Figures**

**
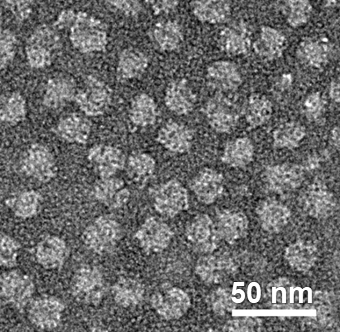
**

**Supplementary Figure 1. Micrograph of negatively stained *E. coli* 70S ribosome.**


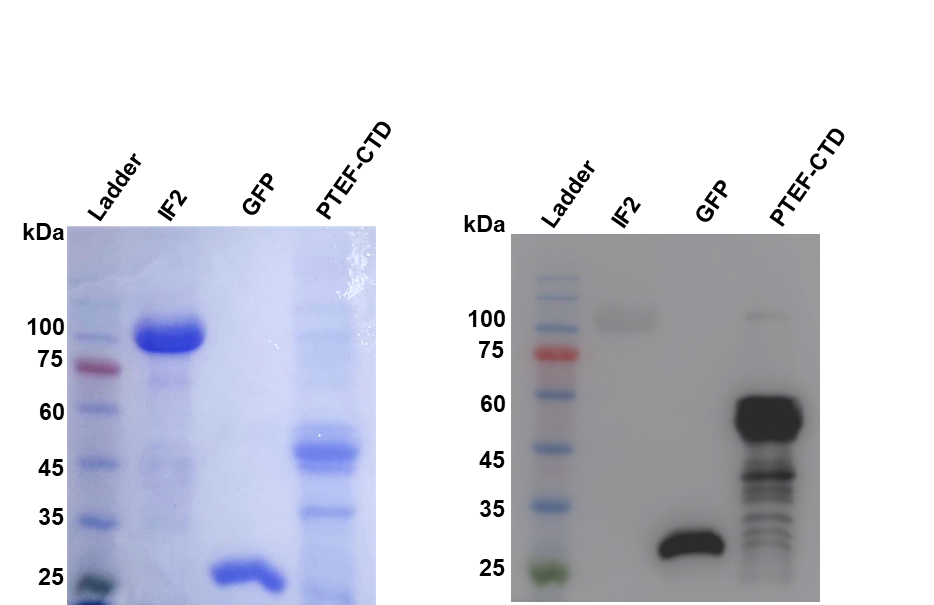


**Supplementary Figure 2. SDS-PAGE (A) and Western blot (B) of *E. coli* IF2, GFP, and PTEF-CTD.** The molecular weights of proteins are *E. coli* IF2 (98 kDa), GFP (28 kDa), and PTEF-CTD (55 kDa) respectively. Anti-His Antibody (MA1-21315, Invitrogen) was used to confirm the presence of the His-tags on the proteins.


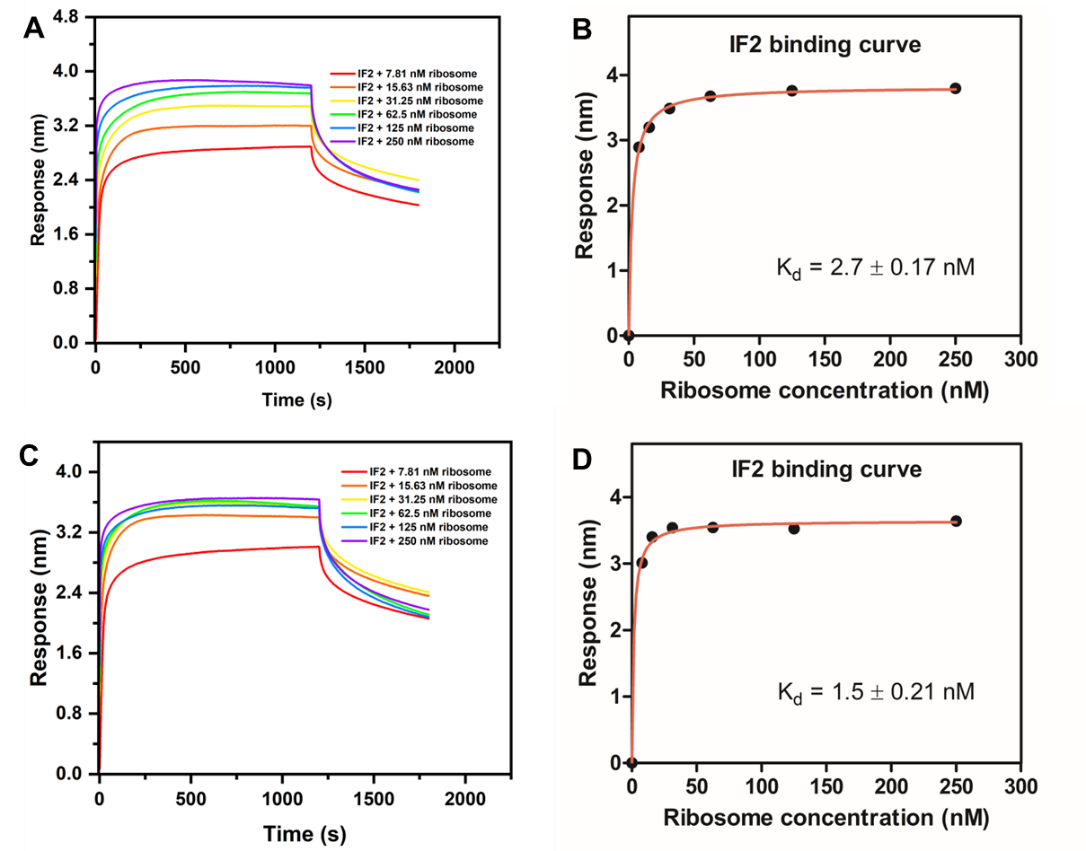


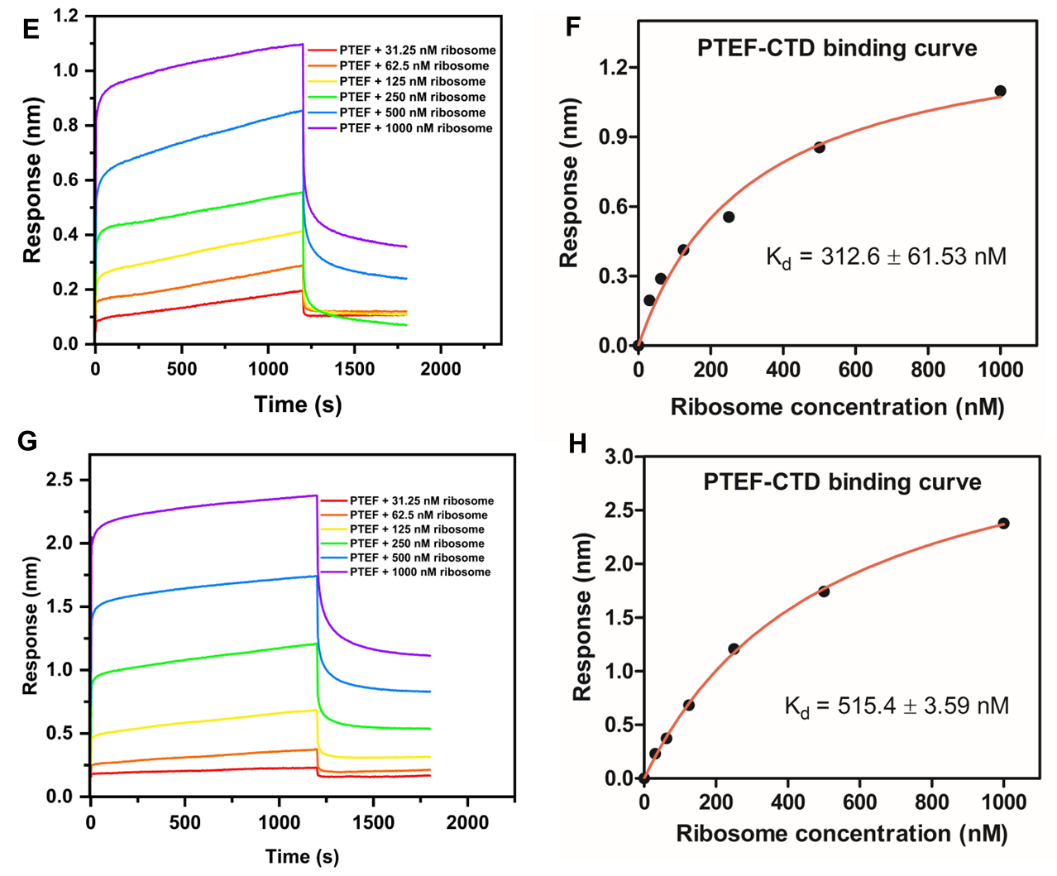


**Supplementary Figure 3. Sensorgram and binding curve for replicate experiments.** Binding sensorgram of *E. coli* IF2 (0.25 µM) (Panel A, C) and PTEF-CTD (0.25 µM) (Panel E, G) with varying *E. coli* 70S ribosome concentrations. Binding affinity curve between *E. coli* IF2 (Panel B, D) and PTEF -CTD (Panel F, H) with *E. coli* 70S ribosome.


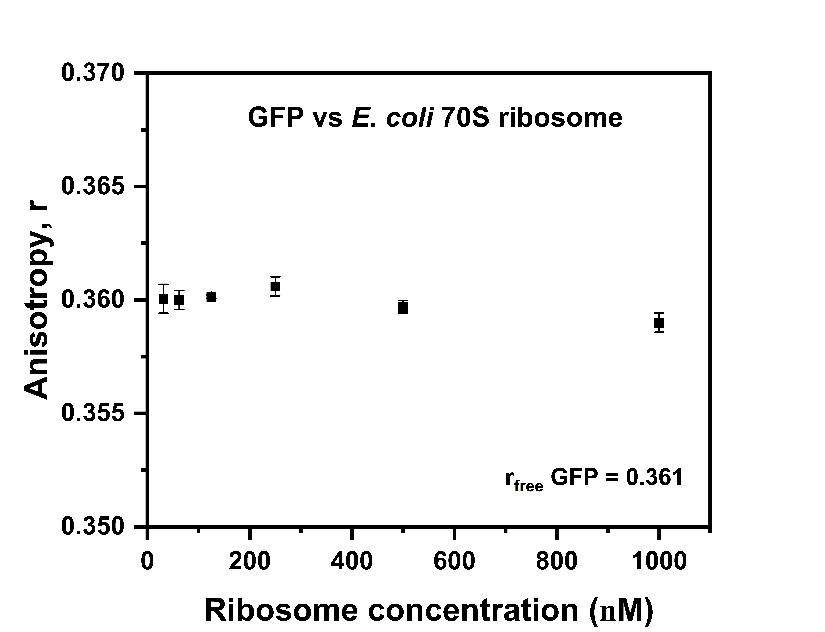


**Supplementary Figure 4. Fluorescence anisotropy between GFP (0.25 µM) vs *E. coli* 70S ribosome.** Data includes varying *E. coli* 70S ribosome concentrations (31.25 nM, 62.5 nM, 125 nM, 250 nM, 500 nM, and 1000 nM). The anisotropy value for free GFP was 0.361.

**Supplementary Table**

**Supplementary Table 1. List of buffer components used.**

| **Buffer name** | **Buffer composition** |
| --- | --- |
| Buffer A | 20 mM Tris HCl pH 7.2, 100 mM NH_4_Cl, 10 mM MgCl_2_, 0.5 mM EDTA and 6 mM βME |
| Buffer B | 20 mM Tris HCl pH 7.2, 500 mM NH_4_Cl, 10 mM MgCl_2_, 0.5 mM EDTA, 37.7% sucrose and 6 mM βME |
| Buffer C | 20 mM Tris HCl pH 7.5, 60 mM NH_4_Cl, 7.5 mM MgCl_2_, 0.5 mM EDTA and 6 mM βME |
| GFP Lysis buffer | 20 mM NaH_2_PO_4_ pH 7.5, 400 mM NaCl, 10 mM Imidazole, 3 mM βME |
| IF2 and GFP Storage buffer | 20 mM HEPES pH 7.5, 100 mM KCl, 10 mM MgCl_2_, 3 mM βME |
| PTEF Storage buffer | 20 mM HEPES pH 7.5, 150 mM NaCl, 10 mM MgCl_2_, 3 mM βME |
